# PW02-025 - Programme necrosis by CAPS-associated NLRP3

**DOI:** 10.1186/1546-0096-11-S1-A166

**Published:** 2013-11-08

**Authors:** T Satoh, N Kambe, H Matsue

**Affiliations:** 1 Dermatology, Chiba University School of Medicine, Chiba, Japan

## Introduction

Cryopyrin-associated periodic syndrome (CAPS), clinically characterized by neutrophil-rich urticarial rash, is associated with missense mutations in *NLRP3*. NLRP3 is a pattern recognition receptor in the cytoplasm of cells and structurally related to plant resistance proteins, which detect pathogen- or danger-associated signals, leading to programmed cell death and hyper response at the local site in plants. In mammals, activated NLRP3 forms inflammasome, which orchestrates the early inflammatory process via IL-1β activation, and also cause programmed necrotic cell death termed “pyronecrosis”. However, the mechanistic details are largely unknown.

## Objectives

To investigate the mechanism of NLRP3-mediated pyronecrosis and its in vivo relevance.

## Methods

We have established a system in which pyronecrosis was induced by the expression of CAPS-associated gain-of-function mutant of NLRP3, using a tetracycline-inducible expression (Tet-on) system. We also induced NLRP3-mediated cell death in mouse air-pouch and harvested the cells and fluid.

## Results

Mutant NLRP3 expression without LPS pretreatment induced only necrotic cell death but not IL-1β secretion in this system. Silencing ASC gene by shRNA prevented pyronecrosis, while silencing caspase-1 did not. When the cell lines expressing NLRP3 mutants by Tet-on system were treated with cathesin B inhibitor, necrotic cell death and speckle patterns of ASC oligomerization were not observed. Interestingly, when these cells were treated with Z-VAD-fmk, the speckle patterns of ASC were seen while they were still alive.

Upon oral administration of doxycycline, injection of LPS-pretreated NLRP3-mutant cells into mouse air-pouch showed necrotic cell death in addition to IL-1β release, resulting in the significant increase in numbers of neutrophils in the pouch. Interestingly, non-pretreated mutant cells, which showed necrotic cell death without mature IL-1β release, also induced neutrophil infiltration, though smaller in number relative to neutrophil infiltration induced by LPS-pretreated mutant cells.

## Conclusion

Pyronecrosis is provoked by downstream of NLRP3-induced ASC oligomerization, but does not require caspase-1 or IL-1β cleavage, and that cathesin B inhibitor and Z-VAD-fmk inhibit pyronecrosis before and after ASC oligomerization, respectively. This clarify the differences of pyronecrosis from pyroptosis which is mediated by ASC oligomerization but do not require NLRP3. In vivo study showed that necrotic cell death by pyronecrosis in itself can cause and exacerbate the neutrophilic inflammatory response.

## Disclosure of interest

None declared.

